# Supplementary material for: The increasing trend in the consumption of ultra-processed food products is associated with a diet related to chronic diseases in Colombia—Evidence from national nutrition surveys 2005 and 2015
Source: PLOS Glob Public Health. 2024 Jan 11;4(1):e0001993. doi: 10.1371/journal.pgph.0001993 (PMC10783756; doi:10.1371/journal.pgph.0001993)
Supplement: S1 Appendix — (DOCX) [file pgph.0001993.s001.docx]

**S1 Appendix.**

The first NOVA group – unprocessed or minimally processed foods – includes fresh, chilled, frozen, or dried fruits and leafy and root vegetables; grains such as brown, parboiled or white rice, corn cob or kernel, wheat berry or grain; legumes such as beans of all types, lentils, chickpeas; starchy roots and tubers such as potatoes and cassava, in bulk or packaged; fungi such as fresh or dried mushrooms; meat, poultry, fish and seafood, whole or in the form of steaks, fillets and other cuts, or chilled or frozen; eggs; milk, pasteurized or powdered; plain yoghurt with no added sugar or artificial sweeteners added; fresh or pasteurized fruit or vegetable juices without added sugar, sweeteners or flavors; grits, flakes or flour made from corn, wheat, oats, or cassava; pasta, couscous and polenta made with flours, flakes or grits and water; tree and ground nuts and other oil seeds without added salt or sugar; spices such as pepper, cloves and cinnamon; and herbs such as thyme and mint, fresh or dried; tea, coffee, and drinking water.

The second NOVA group – processed culinary ingredients – includes salt, sugar, molasses, honey, maple syrup, vegetable oils, butter, lard, starches and other substances extracted from foods or nature and used in home and restaurant kitchens to prepare, season and cook unprocessed or minimally processed foods and to make with them hand-made dishes, soups and broths, breads, preserves, salads, drinks, desserts and other culinary preparations.

The third NOVA group – processed foods – includes canned or bottled vegetables, fruits and legumes; salted or sugared nuts and seeds; salted, cured, or smoked meats; canned fish; fruits in syrup; sugar-coated dry fruits; cheeses; unpackaged freshly made breads, and other relatively simple food products manufactured with the addition of salt or sugar or other substances extracted from foods or nature and of common culinary use to unprocessed or minimally processed foods.

The fourth NOVA group – ultra-processed foods – of greater interest in this study, includes carbonated drinks; sweet or savory packaged snacks; ice-cream, chocolate, candies (confectionery); mass-produced packaged breads and buns; margarines and spreads; cookies (biscuits), pastries, cakes, and cake mixes; breakfast ‘cereals’, ‘cereal’ and ‘energy’ bars; ‘energy’ drinks; milk drinks, ‘fruit’ yoghurts and ‘fruit’ drinks; cocoa drinks; meat and chicken extracts and ‘instant’ sauces; infant formulas, follow-on milks, other baby products; ‘health’ and ‘slimming’ products such as powdered or ‘fortified’ meal and dish substitutes; and many ready to heat products including pre-prepared pies and pasta and pizza dishes; poultry and fish ‘nuggets’ and ‘sticks’, sausages, burgers, hot dogs, and other reconstituted meat products; powdered and packaged ‘instant’ soups, noodles and desserts, and many other ready to consume formulations of several ingredients. Besides salt, sugar, oils, and fats, ingredients of ultra-processed foods include food substances not commonly used in culinary preparations, such as hydrolyzed protein, modified starches and hydrogenated oils, and additives whose purpose is to imitate sensorial qualities of unprocessed or minimally processed foods and their culinary preparations or to disguise undesirable qualities of the final product, such as colorants, flavorings, non-sugar sweeteners, emulsifiers, humectants, sequestrants, and firming, bulking, de-foaming, anti-caking and glazing agents. Unprocessed or minimally processed foods represent a small proportion of or are even absent from the list of ingredients of ultra-processed products.
