# Supplementary material for: The increasing trend in the consumption of ultra-processed food products is associated with a diet related to chronic diseases in Colombia—Evidence from national nutrition surveys 2005 and 2015
Source: PLOS Glob Public Health. 2024 Jan 11;4(1):e0001993. doi: 10.1371/journal.pgph.0001993 (PMC10783756; doi:10.1371/journal.pgph.0001993)
Supplement: Attachment — Submitted filename: RESPONSE_REVIEWERS_V3.pdf [file pgph.0001993.s004.pdf]

Universidad de Antioquia, UdeA Calle 70 No. 52-21, Medellín, Colombia
PhD. Gustavo Cediel
E-mail: gustavo.cedielg@udea.edu.co
November 22, 2023

Response to the reviewers

We appreciate the process and the reviewer's comments to improve the article. The answers to each point are presented below:

Reviewer #1: (No Response)

Reviewer #2: General

First off, I would like to congratulate the authors on the major improvements made to the manuscript, particularly the methods and results section. Nonetheless, some suggestions remain to improve the overall quality of the manuscript.

Response/ thank you. The reviewer comments help to improve the article

Overall, I would suggest to:

• Have someone thoroughly check the English, particularly the construction of some sentences could be improved (some examples provided below).

Response/ someone thoroughly check the English.

• Change the term 'Chronic diseases (CD)-promoting nutrients' to 'Nutrients of concern' which (in my experience) is a more accepted term. In case it is chosen to stick with CD-promoting nutrients or NCD-related nutrients (as in Table 2 and Table 3), make sure to use the same terminology throughout the manuscript. For example, in the discussion also the term critical nutrient is introduced.

Response/ thanks for your comment. In our experience, "Nutrients of concern", include both 'chronic diseases (CD) related nutrients (free sugars, trans fats, total fats, saturated fats, sodium, etc) and health promoting nutrients such potassium, fiber, calcium, etc. Given that this article is focused in CD-related nutrients, we prefer to be clear and use this term in the document.

• Improve the relation between the introduction and the discussion. Both currently still feel a little all over the place, especially the discussion. I would recommend to choose the core topics you want to discuss in both sections and double check that no new topics are discussed in detail in the discussion section which have not been introduced in the introduction.

Response/ thanks for your comment. We made the change

Abstract

• The abstract would benefit from some reorganisation. Currently it still lacks a little information in the background section while it goes very much into detail in the results section. I would recommend to really focus on the key findings.

Response/ thanks for your comment. We made the change

• In the conclusion of the abstract it is stated that 'the increasing trend in the consumption of UPF is associated with diet-related CD'. This isn't a conclusion that can be made solely on these results as this study did not look at CD, solely at nutrients of concern/ CD-promoting nutrients. It would be good to formulate a more accurate and to the point conclusion.

Response/ thanks for your comment. We made the change

Introduction

• Line 66-67: 'The Pan American Health Organization (PAHO) reported a growing trend in the sales of these products in Latin America' -> Change 'these products' to 'UPFs'. Remove the comma at the end of the sentence (after 2013).

Response/ thanks for your comment. We made the change

• Line 67-68: 'In Colombia, it was similar, being 25%, the gain for the same period' -> Change to something in the lines of 'In Colombia a similar increase of 25% over the same period was observed'.

Response/ thanks for your comment. We made the change

• Line 70-71: 'The highest consumption was observed among adolescents, contributing 20.3% to the total daily energy consumed' -> Change to something in the lines of 'The highest consumption was observed among adolescents, with UPF contributing 20.3% to the total daily energy intake'.

Response/ thanks for your comment. We made the change

• Line 80-81: The last sentence linking to the corporative food regime appears rather suddenly. Maybe add some literature about the link between the corporate food regime and the increased sales of UPFs. The same goes for the next sentence (line 84-87) which could be in the same paragraph as the previous one. As this aspect is also thoroughly discussed later on it would make sense to allocate one paragraph on the food industry in the introduction.

Response/ thanks for your comment. We made the change

Methods

• Overall, I would recommend to make it clear which explanation is about the work that the authors/researchers did themselves and which parts are rather an explanation about existing databases (ENDS and ENSIN).

Response/ thanks for your comment. We made the change

• Line 98: Idem as for ENSIN between brackets, change '(ENDS, 2005, 2015)' to '(ENDS 2005 and 2015)'.

Response/ thanks for your comment. We made the change

• Line 103-112: From how the paragraph is currently formulated it still seems as if the authors/researchers collected the dietary data, while these data seem to be obtained from ENSIN. Still assuming that this is about how the ENSIN data were collected, I suggest to make this more clear in this paragraph (e.g. start the paragraph with 'The dietary data within ENSIN were obtained using….').

Response/ thanks for your comment. We made the change

• Line 119: Currently it remains unclear how these 33 subgroups were decided upon. Is it based on a certain classification? Based on how the ENSIN database is organised?

Response/ thanks for your comment. We made the change in 142

• Line 130-131: 'The nutritional information from packaged foods was collected in significant supermarket chains in Colombia.' -> Collected by who is in charge of the Colombian food composition table or by the authors/researchers? Moreover, what is meant by significant supermarket chains? Significant because of their market share in

general in the country? Or because of there presence in bigger cities? How were the supermarkets selected?

Response/ thanks for your comment. We made the change. It was a typo error; “significant” was a wrong translation.

• Line 136: Add fats after trans (~trans fats).

Response/ thanks for your comment. We made the change

• Line 140: Do you mean the sociodemoDSgraphic information available within ENDS? If sociodemographic information was already available in ENSIN, for what exactly was the ENDS information used?

Response/ thanks for your comment. We made the change. It was in ENDS

• Line 146-147: ‘The collected data was classified into one of four levels.’ -> Four levels of what?

Response/ thanks for your comment. We made the change. It was a typo error.

• Line 162: Remove bracket after ingredients.

Response/ thanks for your comment. We made the change

Results

• Figures: The current figures (Figure 1 and Figure 2) as attached to the manuscript need to be sharpened to make them readable.

Response/ thanks for your comment. We made the change

• Tables: Just a minor detail, but it would be nicer to have the legend in order of appearance of the symbols in the table.

Response/ thanks for your comment. We made the change

• Line 249-252: Reorganise this sentence to something like: Making a combination of groups to evaluate a proxy of consumption behavior by dietary patterns, a decrease in the consumption of culinary preparations (-12.7%, combining energy intake from unprocessed or minimally processed foods and processed culinary ingredients) is observed between 2005 and 2015.

Response/ thanks for your comment. We made the change

• Line 261-268: In the discussion it is mentioned 'that people aged below 19 years, in the higher wealth index quintiles, living in urban areas, and without ethnicity or black/mulato/afro-descendant, according to the self-report, were the primary consumers of UPF'. In the current manuscript this result doesn't seem to be reported in the result section yet. Maybe something about this could be added to this paragraph?

Response/ thanks for your comment. The result has been reported in the result section in line 286.

• Line 267: Most inferior wealth status sounds a little condescending. I would suggest to formulate this in a more neutral way (potentially something like 'from the lower wealth index quintiles' or 'had a lower wealth status').

Response/ thanks for your comment. We made the change

• Line 292: As earlier the abbreviation PAF was introduced for population attributable fractions, maybe stick to using this same abbreviation?

Response/ thanks for your comment. We made the change

• Line 292-293: Change 'inadequate intake' to 'excessive intake'?

Response/ thanks for your comment. We made the change

Discussion

• Overall, I would recommend to discuss the results more thoroughly. Currently there are a lot of longer lists with characteristics and suggestions for regulations in comparison to the extent in which the results are discussed.

Response/ thanks for your comment. We made the change

• The first paragraph of the discussion provides a very nice summary!

Response/ thanks for your comment.

• Line 309-311: Change to 'would be reduced to'. Moreover, I would suggest to use the same expression for all the nutrients (fractions or percentages).

Response/ thanks for your comment. We made the change

• Line 320: Here a new terminology is introduced ~ 'critical nutrients' instead of 'CD-promoting nutrients'. Stick to the same terminology throughout the manuscript.

Response/ thanks for your comment. In our experience, "Nutrients of concern", include both 'chronic diseases (CD) related nutrients (free sugars, trans fats, total fats, saturated fats, sodium, etc) and health promoting nutrients such potassium, fiber, calcium, etc. Given that this article is focused in CD-related nutrients, we prefer to be clear and use this term in the document.

• Line 325-332: This is a result from the study, so important to add this to the result section (for example to paragraph line 261-268; see suggestion above).

Response/ thanks for your comment. We made the change

• Line 331-332: Maybe simplify this last sentence to something like 'These findings are in line with the ENSIN 2005 results (ref)'.

Response/ thanks for your comment. We made the change

• Line 334: Remove 'alarming', or, as I do agree that it is kind of alarming, explain better to the reader why this situation is alarming. This could be done, for example, by adding some more literature on the risks of UPFs (~In light of the substantial amount of recent research accentuating the risks of UPFs (references), the results presented above reflect an alarming situation which has been associated with the corporate food regime…'.

Response/ thanks for your comment. We made the change

• Line 335: Since-> in (emerged in the 1990s).

Response/ thanks for your comment. We made the change

• Line 345: Can remove 'in the population'.

Response/ thanks for your comment. We made the change

• Line 345-346: Minor detail - before the different characteristics didn't start with a capital letter and from (viii) onwards they do. I would recommend to be consistent.

Response/ thanks for your comment. We made the change

• Line 354: Remove mainly.

Response/ thanks for your comment. We made the change

• Line 356-359: This is very true and highly important, but within the discussion I would stick to discussing issues related directly to the findings of this study.

Response/ for us, it is essential to include this kind of issue in the discussion because it is related to the study results and also because this is evidence that our group has been created intentionally to explain socially the phenomena of increasing UPs in the country.

• Line 365-369: As Martinez and Cols found similar results, including for Colombia, it might be good to add in the introduction what the added value is of this study compared to theirs.

Response/ thanks for your comment. We made the change

• Line 367: The questions that immediately came to mind was, which eight countries. Maybe add between brackets?

Response/ thanks for your comment. We made the change

• Line 371: Maybe slightly reformulate this sentence? For example 'The results of this study highlight the need for regulations of UPFs…'.

Response/ thanks for your comment. We made the change

• Line 371-398: I would recommend to stick to suggesting regulations related directly to the results of this study. For example, I completely support the recommendation to implement warning labels, but this isn't directly related to UPFs. In line with this I also completely agree with suggestion (iv), but this isn't really a policy recommendation that can be made based on the current results (could be if this becomes a more important focus of the discussion, but that currently isn't the case).

Response/ for us, it is essential to include this kind of issue in the discussion because it is related to the study results and also because this is evidence that our group has been created intentionally to explain socially the phenomena of increasing UPs in the country

• Line 377: Change to 'where they have these recommendations:…' (without capital letter).

Response/ thanks for your comment. We made the change

• Line 404: Maybe use 'nonetheless' instead of 'however'?

Response/ thanks for your comment. We made the change

Conclusions

• Overall I would recommend to keep the conclusion shorter and more focussed on the true results of the study itself.

Response/ thanks for your comment. We made the change

• Line 430-433: In my opinion this would better fit under the discussion as this is not a results of the study itself.

Response/ thanks for your comment. We made the change

• Line 433: Potentially reformulate to something like 'These data highlight the need to improve the current health promotion and prevention policies….'.

Response/ thanks for your comment. We made the change
