# Supplementary material for: The increasing trend in the consumption of ultra-processed food products is associated with a diet related to chronic diseases in Colombia—Evidence from national nutrition surveys 2005 and 2015
Source: PLOS Glob Public Health. 2024 Jan 11;4(1):e0001993. doi: 10.1371/journal.pgph.0001993 (PMC10783756; doi:10.1371/journal.pgph.0001993)
Supplement: Attachment — Submitted filename: Responses to the journal_GCed.docx [file pgph.0001993.s003.docx]

**Medellín, August 5, 2023**

**Dear Editor and reviewers**

**We appreciate your comments. The responses to the journal requirements and the reviewers are presented at each point bellow.**

**Best regards**

**Gustavo Cediel. PhD**

**University of Antioquia**

**gustavo.cedielg@udea.edu.co**

************************************************************************

Journal Requirements:

1. We noticed you have some minor occurrence of overlapping text with the following previous publication(s), which needs to be addressed:

- 10.1017/S1368980019004737
- <https://doi.org/10.11606/s1518-8787.2020054001176>

In your revision ensure you cite all your sources (including your own works), and quote or rephrase any duplicated text outside the methods section. Further consideration is dependent on these concerns being addressed.

**Response/ Thanks for your comment. The text was edited.**

2. Please ensure that Funding Information and Financial Disclosure Statement are matched.

**Response/ Thanks for your comment. The information was included.**

3. In the Funding Information you indicated that no funding was received. Please revise the Funding Information field to reflect funding received.

**Response/ Thanks for your comment. The information was included.**

4. Please provide separate figure files in .tif or .eps format only and remove any figures embedded in your manuscript file. Please also ensure all files are under our size limit of 10MB.

For more information about figure files please see our guidelines:

<https://journals.plos.org/globalpublichealth/s/figures> 
<https://journals.plos.org/globalpublichealth/s/figures#loc-file-requirement>

**Response/ Thanks for your comment. The figures files were changed to the JPG format.**

5. We have noticed that you have uploaded Supporting Information files, but you have not included a list of legends. Please add a full list of legends for your Supporting Information files after the references list.

**Response/ Thanks for your comment. The list of legends was included after the references list.**

**Responses to the Reviewer #1:**

I would like to thank the Editor and authors for the opportunity to review this manuscript. The topic is very relevant due to the increased concern of the role of ultra-processed foods in the nutrition transition underway, particularly in low and middle-income countries. The authors analysed data from a nationally representative sample of Colombians, providing first time evidence of trends using individual-level consumption data. Some suggestions to improve the quality of the manuscript are presented below:

**General comments:**

- The manuscript needs proofreading throughout (e.g., line 148).

**Response/ the manuscript was proofread**

- It is unclear how objectives ii and iii add to previous evidence using 2005 data. The originality and relevance of new analysis should be explained further in the introduction and discussion; e.g., the rising in UPF consumption could explain changes in prevalence of inadequate intake. For that end, instead of presenting aims ii and iii only with 2015 that, would be interesting to see changes in prevalence between 2005-15 attributable to increased intake of UPF. For instance, following this methodology: doi:10.3389/ijph.2022.1604103

**Response/ Thanks for your comment. We added additional arguments in the discussion regarding the relevance of this work (Line 409). Objectives ii) and iii) correspond to the last national representative database in Colombia (2015), The comparison with the previous national representative analysis is possible in terms of dietary consumption (by NOVA classification), but we think is not possible in terms of nutrient content, because, some critical nutrients are not available in 2005. For the same reason it is not possible to use the methodology of Louzada et al.**

**.**

- Table 1 requires t-test of mean differences to state that there are significant temporal variance.

**Response/ Thanks for your comment. The analysis was included.**

- Figure 1 is a good visual, but would be better if accompanied by a table (could be supplementary material) presenting coefficients of associations (and respective 95%CI or p-values) of UPF consumption among different strata of the population. Similarly, would be good to present the variation in UPF consumption between 2005-2015 in different sociodemographic groups.

**Response/ Thanks for your comment. The analysis was included in supplemental material**

- Although methods and discussion mention the use of standardized coefficients, these do not seem to be presented in Table 2.

**Response/ thanks for your comment. The real analysis was beta regression coefficients, we change this error in the document.**

- Could you please provide as supplementary material or in the main manuscript beta coefficients of the association of UPF consumption continuous and CD-nutrient intakes of Table 2? This is useful because quintile 5 has a large variability in UPF intake (33 to 100%).

**Response/Table 2 presents crude and adjusted models and beta regressions of the association between ultraprocessed foods and consumption of CD nutrients intakes**

- Figure 2 methods, results and discussion: if the level represented is the one among the 20% lowest consumers, UPF intake was then set to the upper bound (4.5%) instead of 1%?

**Response/ Thanks for your comment. We added details of the population attributable fractions analysis in Methods Line 183.The population attributable fractions (PAFs) were calculated to estimate the reduction in the prevalence of nutrient inadequacies if the consumption of ultra-processed foods in Colombia was the one seen in the 20% lowest consumers of ultra-processed foods (the first quintile). The dietary share of ultra-processed foods among these consumers (n=6820) represented 1.0% of total energy intake ranging from 0% to 4.5%.**

- Discussion is lacking explanation why more advantaged people have higher intakes of UPFs in Colombia.

**Response/ Thanks for your comment. We added details of this explanation in Line 325.**

Minor comments:

Abstract:

- Methods: replace “degree and extent” with “extent and purpose”

**Response/ thanks for your comment. The change was made**

- Results: Please provide p-value considered significative

**Response/ thanks for your comment. The change was made**

- Conclusions: Missed comments related to the time trends and implications of UPF to the decreasing in the quality of diets in Colombia.

**Response/ thanks for your comment. The change was made**

Line 138: sociodemographics: missing rural/urban residence and description of pregnant women presented in Fig 1. It is unclear why pregnant women were included in Fig 1.

**Response/ thanks for your comment. The change was made**

Table 2: Sodium is mg/day or density?

**Response/ mg/day, given that recommendations are**

Table 3: ** p<0.05 can be removed

**Response/ thanks for your comment. The change was made**

Line 78: missing “respectively” in the end of the sentence

**Response/ thanks for your comment. The change was made**

Line 80: UPF consumption instead of use?

**Response/ thanks for your comment. The change was made**

Line 82: Would be good to provide evidence on the prevalence of chronic diseases (e.g., obesity) in Colombia, particularly if there was growth.

**Response/ thanks for your comment. The provide a reference 5 in line 62**

Line 115: extent and purpose? Keep it consistent throughout.

**Response/ thanks for your comment. The change was made**

Line 118: subgroups within the NOVA 4 groups?

**Response/ thanks for your comment. The change was made**

Line 103: age >1y instead?

**Response/ thanks for your comment. The change was made**

Thank you very much again for the opportunity to review this manuscript.

**Response/ thank you very much for all your comments. These help to improve the manuscript**

**Reviewer #2: General**

First off, I would like to congratulate the authors on the interesting study they conducted. Nonetheless, the manuscript would benefit from being thoroughly revised as to improve the strength and readability.

• At times the language is unclear, making it difficult to follow. I would advise the authors to work with a native English speaker or English speaking copyeditor to improve the readability.

**Response/ Response/ Thanks for your comment. The text was edited.**

• The term ‘Chronic diseases (CD)-promoting nutrients’ is used throughout the text to refer to sugar, fat (total, saturated and trans) and sodium. This seems a strong statement to me. There is a strong link between diets and chronic diseases, but is there sufficient evidence to support a direct link with nutrients?

**Response/ thanks for your comment. Our references in the term Nutrient related to Chronic diseases are: i) the Nutrient profile model to prevent chronic disease from PAHO, and ii) the manuscript of Diet, nutrition and the prevention of chronic diseases. Report of a joint FAO/WHO Expert Consultation (references 6 and 7).**

**Reference 6. Pan American Health Organization. Nutrient Profile Model [Internet]. Washington, D.C.: PAHO; 2016 [cited 2017 Mar 1]. p. 1–31. Available from: http://iris.paho.org/xmlui/handle/123456789/18621**

**Reference 7. World Health Organization. Diet, nutrition and the prevention of chronic diseases. Report of a joint FAO/WHO Expert Consultation. World Heal Organ Tech Rep Ser [Internet]. 2003 [cited 2017 Dec 13];916:61–71. Available from: http://www.who.int/dietphysicalactivity/publications/trs916/en/**

• The introduction needs to be strengthened. Currently it provides numerical information, but does not clearly lead up to the objectives. For example, in the discussion the corporate food regime and corporate political activity are brought up. It could be interesting to already add this in the introduction leading up to the objectives of the study.

**Response/ thanks for your comment. The recommendation was included in the introduction in line 80**

• Methods needs to be more clear and to the point:

**Response/ / Thanks for your comment. The text was edited.**

o Make a more clear distinction between what you as researchers/authors did and what was obtained from existing surveys. For example, ENSIN data were 24-hour recall data, but when in the text describing these data it sometimes seems these were collected by the researchers

**Response/ Thanks for your comment. The text was edited.**

o When reading the results it says ‘making a combination of groups with the aim of evaluating a proxy of consumption behavior by dietary patterns…’. It would be beneficial to already describe what was done, why and how in the methods section.

**Response/ thanks for your comment. The recommendation was included in the methods in line 159**

• The current results section often describes in rather lengthy detail what is shown in the table. In my opinion this section could benefit from in text solely writing the important results and referring to the tables for the details.

**Response/ Thanks for your comment. The text was edited.**

• Although there is a lot of very interesting information within the current discussion, it feels a little all over the place. I would recommend to focus on discussion points directly stemming from the results from the study and placed within the context introduced at the beginning (within the introduction).

**Response/ Thanks for your comment. The text was edited.**

Below I have included some thoughts that came to mind when reading the different sections, which might help clarify some of the issues mentioned above.

**Response/ Thanks for your comments.**

**Abstract**

• Tine layout remark: I would start each section (e.g. objectives, methods,…) on a new line to create a better overview.

**Response/ thanks for your comment. The change was made**

• In the beginning, maybe add at least one introductory sentence. Just listing the objectives feels like a strange beginning.

**Response/ thanks for your comment. The change was made**

• Line 36-37: There is a strong link between diets and chronic diseases, but is there sufficient evidence to support the claim ‘…CD-promoting nutrients such as…’. In my opinion this might be too strong a statement.

**Response/ Thanks for your comment. Our references in the term Nutrient related to Chronic diseases are: i) the Nutrient profile model to prevent chronic disease from PAHO, and ii) the manuscript of Diet, nutrition and the prevention of chronic diseases. Report of a joint FAO/WHO Expert Consultation (references 6 and 7).**

• Line 39-40: ‘excessive intake of all CD-promoting nutrients (according to WHO recommendations)’ - CD-promoting nutrients according to WHO or nutrients above the thresholds defined by WHO? Not 100% clear to me.

**Response/ Thanks for your comment. Same that the previous point. Our references in the term Nutrient related to Chronic diseases are: i) the Nutrient profile model to prevent chronic disease from PAHO, and ii) the manuscript of Diet, nutrition and the prevention of chronic diseases. Report of a joint FAO/WHO Expert Consultation (references 6 and 7).**

**Introduction**

• Apart from the first paragraph, the current introduction provides a lot of interesting numerical information, but does not clearly lead up to the objectives. The introduction could be made stronger by making it more clear why this study is so important within the current setting and context.

**Response/ Thanks for your comment. The text was edited.**

• Line 65-66: ‘…these products in Latin America, with a 26% increase between the year 2000 and 2013, being 25%, the increase in Colombia for the same period.’ Not completely clear. Do you mean that the trend in Colombia was similar with a 25% increase over the same time period? If so, split up into two sentences.

**Response/ Thanks for your comment. The text was edited.**

• Line 67: remove from (‘national level from in 2005-> national level in 2005)

**Response/ Thanks for your comment. The text was edited.**

• Line 68: change to ‘came from UPF’ – was in the past (2005).

**Response/ Thanks for your comment. The text was edited.**

• Line 66-70. Sentence is rather long. Maybe split up into two sentences to make it more clear? E.g. ‘Additionally, analysis of food consumption at the national level in 2005 showed that 15.9% of energy consumed by the population of Colombia came from UPF. The highest consumption was observed among adolescents with a contribution of 20.3% to the total energy consumed per day.’

**Response/ Thanks for your comment. The change was made.**

• Line 72: Move ‘in Colombia to after ‘all types of UPF’.

**Response/ Thanks for your comment. The change was made.**

• Line 73-74: Frozen products, canned products, preserved products and dairy products aren’t necessarily ultra-processed.

**Response/ Thanks for your comment. The text was edited.**

• Line 76-78: Is this overarching for Brazil, Chile and Mexico? Or respectively for each country?

**Response/ Thanks for your comment. In each country. The text was edited.**

• Line 76: comma before such as.

**Response/ Thanks for your comment. The change was made.**

• Line 77: comma after Mexico.

**Response/ Thanks for your comment. The change was made.**

• Line 78: of-> with

**Response/ Thanks for your comment. The change was made.**

• Line 82-85: Apart from the first sentence, this paragraph does not clearly lead up to the objectives.

**Response/ Thanks for your comment. The text was edited.**

o Nothing has been mentioned before on foreign investment within the introduction.

**Response/ Thanks for your comment. The text was edited, included the topic of corporative food regime, as the reviewer mentioned in a previous point**

o Nothing has been mentioned on overweight and obesity in Colombia within the introduction.

**Response/ thanks for your comment. The provide a reference 5 in line 62**

o Apart from the first paragraph, the introduction does not reflect well the issue of UPF.

**Response/ Thanks for your comment. The text was edited.**

• In the discussion (line 368-372) the study of Martinez and cols is mentioned which was conducted across 8 countries, including Colombia, and had similar findings. This immediately made me wonder about the added value of this study. There definitely is an added value as you had data from 2005 and 2015 and (from the current text), seem to have gone more into depth, but this needs to be made more clear in the introduction leading up to objectives.

**Response/ Thanks for your comment. We added additional arguments in the introduction and discussion regarding the relevance of this work (Line 409)**

**Methods**

• Line 94-95: remove ‘y’ after the dates in brackets.

**Response/ Thanks for your comment. The change was made**

• Line 96-97: between the brackets change to ‘ENSIN 2005 and 2015’.

**Response/ Thanks for your comment. The change was made**

• Line 102-111: From this paragraph it sounds as if the authors did this, but in the previous paragraph it says that the data from ENSIN were used for this. Assuming that this is about how the ENSIN data were collected, reformulate the paragraph to make this clear.

**Response/ Thanks for your comment. The text was edited.**

• Line 118: 33 subgroups? Only explained the NOVA classification above (with 4 groups), so please explain what is meant here.

**Response/ Thanks for your comment. The text was edited in line 159. These 33 groups are classified within the NOVA 4 groups**

• Line 118-123: Not clear what is meant. Please clarify.

**Response/ Thanks for your comment. In 5.6% of the cases, it was not possible to break down the typical culinary preparations into their constituent ingredients (for example, "Cooked lasagna pasta", "Arepa de chocolo", "Tamal") and were classified in NOVA group 1 as minimally processed foods in the 'freshly prepared food' category and those ready-to-eat fried, sweet or savory preparations were classified as processed foods NOVA group 3 (eg, “buñuelos”, “desserts”, “fried empanadas”).**

• Line 126: energy content.

**Response/ Thanks for your comment. The change was made**

• Line 126: what type of sugars? Added, total,…?

**Response/ Thanks for your comment. It is free sugars. The change was made**

• Line 132: ‘were used for evaluating the prevalence of non-recommended intake of different key nutrients’ -> Were used to determine the prevalence of excessive intake of nutrients of concern? Non recommended intake can also be too little, while I assume the focus here is on too much.

**Response/ Thanks for your comment. The change was made**

• Line 138-145: About extra data collected by authors or about the data available within ENDS?

**Response/ Thanks for your comment. The change was made**

• Line 147-154: Improve this paragraph. Currently there is quite some rather vague information there.

**Response/ Thanks for your comment. The text was edited.**

• Line 159: CD-promoting nutrients seems like a too strong choice of words. Maybe rather something in the lines of ‘nutrients of which excessive intake according to the WHO thresholds has been linked to CD?

**Response/ Thanks for your comment. The change was made**

• Line 165: Minor questions, but why did you chose for quintiles?

**Response/ there is no thresholds of adequate intake of ultraprocessed foods. Quintiles as a variable polychromous allow establishing an order of degree as better or worse, higher or lower, or more or less.**

• Line 176-177: ‘reduction in the prevalence of nutrient inadequacies’ -> Inadequate sounds like people aren’t getting enough, but I suppose that you want to say the opposite.

**Response/ Thanks for your comment. The change was made by “excessive intake of nutrients that according to the WHO thresholds has been linked to CD”**

Results

• The text currently repeats a lot what is already shown in Table 1 (e.g. line 200-232). In the text I would suggest to focus on the overarching findings that are interesting and reference to Table 1 for the details.

**Response/ Thanks for your comment. The text was edited.**

• Table 1: the ‘se’ in brackets after the mean, does this refer to the standard deviation? If so, please clarify in the title.

**Response/ Response/ Thanks for your comment. The change was made. It is standard error**

• Line 200-201: ‘Table 1 also shows the contribution of different subcategories of products within the NOVA categories to energy and nutrients intake’ – Within the current table I only see the contribution to energy, not nutrients.

**Response/ Thanks for your comment. The change was made**

• Line 237: In text says -4.2% while in the table it says -4.1%. Check which one is correct and adjust.

**Response/ Thanks for your comment. The change was made in the text**

• Line 241: Making a combination of groups? Please add some information on this in the methods section

**Response/ Thanks for your comment. The change was made in methods in line 159**

• Figure 1: Currently the numbers are rather small. Maybe increase the overall figure?

**Response/ Thanks for your comment. The change was made**

Discussion

• The current discussion doesn’t always flow naturally from the results that were really found during this study. E.g. on line 319 it suddenly mentions ‘unsustainable’ which is less relevant for this article and these findings.

**Response/ Thanks for your comment. The text was edited.**

• Line 334: ‘in analysis from 2005’. Which analysis? Please provide some more information.

**Response/ Thanks for your comment. The change was made in the text**

• Line 336-349: In the introduction the corporate food regime was hardly mentioned or introduced. This makes it very strange to then here have such a long paragraph about this.

**Response/ Thanks for your comment. We added additional information in the introduction about the corporative food regime in Line 80**

• Line 355-362: Same as for the above. The Corporative Political Activity (CPA) was not yet touched upon in the introduction, which makes it a little strange to have this here. Both corporate food regimes and CPA could however be very interesting to include in the introduction if you feel this is an important reason for doing this study and context for the results.

**Response/ Thanks for your comment. We added additional information in the introduction about the corporative food regime in Line 80**

• Line 376: Law 2120 – Please provide some context. Most readers (like me) won’t know this law.

**Response/ Thanks for your comment. We clarify in the discussion that law 2120 refers to improving food environments to prevent CD. Here a link with more information.**

**https://www.camara.gov.co/asi-sera-la-promocion-de-entornos-alimentarios-saludables-y-prevencion-de-enfermedades-no**

• Line 378-379: Shortly explain the examples of Brazil and Uruguay

**Response/ We added additional information in the discussion about the recommendation of these dietary guidelines in Line 375**

• Line 378: Apply the PAHO nutrient profile model for what? Restricting marketing? Accessibility of products?

**Response/ Thanks for your comment. The change was made in the text in line 381**

• Line 394-396: Although I completely agree, I don’t see how this recommendation flows from the results of this study.

**Response/ Thanks for your comment. The text was edited.**

• Line 398: Was collected? This gives the impression that this was done within this study, but earlier I had understood that this were the ENSIN data. Please be specific about this.

**Response/ Thanks for your comment. The change was made**

Conclusions

• The conclusion could be a little more focused on the results itself and the most important conclusions. Currently it still feels a little too much like a continuation of the discussion.

**Response/ Thanks for your comment. The text was edited.**
